# Molecular Optical Imaging with Radioactive Probes

**DOI:** 10.1371/journal.pone.0009470

**Published:** 2010-03-01

**Authors:** Hongguang Liu, Gang Ren, Zheng Miao, Xiaofen Zhang, Xiaodong Tang, Peizhen Han, Sanjiv S. Gambhir, Zhen Cheng

**Affiliations:** 1 Molecular Imaging Program at Stanford, Department of Radiology and Bio-X Program, Stanford University, Stanford, California, United States of America; 2 Department of Bioengineering, Stanford University, Stanford, California, United States of America; 3 Department of Physics, University of Notre Dame, Notre Dame, Indiana, United States of America; 4 Institute of Radiation Medicine, Chinese Academy of Medical Sciences, Peking Union Medical College, Tsinghua University, Tianjin, People's Republic of China; Genentech, United States of America

## Abstract

**Background:**

Optical imaging (OI) techniques such as bioluminescence and fluorescence imaging have been widely used to track diseases in a non-invasive manner within living subjects. These techniques generally require bioluminescent and fluorescent probes. Here we demonstrate the feasibility of using radioactive probes for *in vivo* molecular OI.

**Methodology/Principal Findings:**

By taking the advantages of low energy window of light (1.2–3.1 eV, 400–1000 nm) resulting from radiation, radionuclides that emit charged particles such as β^+^ and β^−^ can be successfully imaged with an OI instrument. *In vivo* optical images can be obtained for several radioactive probes including 2-deoxy-2-[^18^F]fluoro-D-glucose ([^18^F]FDG), Na^18^F, Na^131^I, ^90^YCl_3_ and a ^90^Y labeled peptide that specifically target tumors.

**Conclusions/Significance:**

These studies demonstrate generalizability of radioactive OI technique. It provides a new molecular imaging strategy and will likely have significant impact on both small animal and clinical imaging.

## Introduction

Molecular imaging is a relatively new yet fast growing research discipline. Practical molecular imaging enables researchers to study diseases non-invasively in living subjects at the molecular level [1], [2], [3], [4]. Numerous studies have demonstrated that molecular imaging techniques play a central role in the era of personalized medicine. A variety of imaging modalities have been developed that provide functional and anatomical information of diseases in living small animals and patients. Such modalities include positron emission tomography (PET), single photon emission computed tomography (SPECT), optical imaging (OI, bioluminescence and fluorescence), magnetic resonance imaging (MRI), ultrasound (US), and computed tomography (CT) [1], [2].

OI has rapidly gained popularity in molecular imaging. It is an inexpensive imaging technique due to the low costs of detection devices. The technique is easy to learn and use and interpretation of the images is generally straightforward. Imaging in the near-infrared (NIR) region has advantages of improved tissue penetration and low tissue autofluorescence which enhances target to background ratios [5]. While OI generally detects low energy light (visible or near-infrared light) emitted from bioluminescence or fluorescence probes, radioactive molecular probes are traditionally imaged with PET, SPECT or gamma (γ) cameras that detect high energy γ rays [1]. We hypothesized that radionuclide radiation in the low energy window of light (1.2–3.1 eV, 400–1000 nm) could be imaged using OI techniques and be especially valuable for molecular OI. Thus we examined a variety of radionuclides with different types of emission properties (β^+^, β^−^ or γ) using a commercially available OI instrument for *in vitro* and *in vivo* optical imaging. During the preparation of this manuscript, Robertson et al. successfully demonstrated that high-energy β^+^ emitters, ^18^F and ^13^N, could be used for optical imaging [6]. This pioneer work provides a solid foundation of using PET radioisotopes for optical imaging. Our study further evaluated a variety of radionuclides (β^+^, β^−^ and γ emitters) for optical imaging. *In vivo* optical images can be obtained for several radioactive probes such as 2-deoxy-2-[^18^F]fluoro-D-glucose ([^18^F]FDG), Na^18^F, Na^131^I, ^90^YCl_3_ and a ^90^Y labeled tumor targeting peptide. The results presented here bridge the subfields of imaging by visualizing radioactive probes with OI. Our study demonstrates the feasibility of molecular imaging of living subjects using OI modalities in conjunction with a wide diversity of radioactive probes.

## Results

### Radioactive Materials That Can Be Used for OI

**Figure 1:**
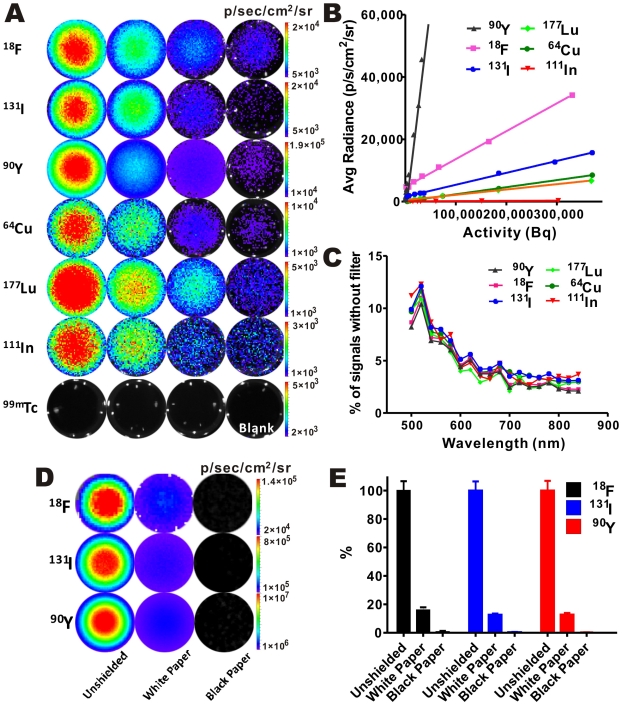
Optical signals are detectable by OI instruments and have a continuous spectrum. (**A**) Most nuclides, except ^99m^Tc, provide OI signals with sensitivity within as low as 0.004–0.370 MBq (0.1–10 µCi) range. (^18^F: 5, 2, 1, 0.1 µCi; ^131^I: 10, 5, 1, 0.1 µCi; ^90^Y: 5, 1, 0.2, 0.01 µCi; ^64^Cu: 10, 5, 1, 0.1 µCi; ^177^Lu: 10, 5, 2, 0.5 µCi; ^111^In: 10, 5, 1, 0.5 µCi; ^99m^Tc: 20, 10, 5 µCi ). (**B**) Detection sensitivity of different radionuclides. Radionuclides with a higher *K* value have stronger signal intensity. (**C**) Similar spectra were ovserved from different radionuclides. Results are presented as the ratio of photons detected using a narrow band emission filter (20nm bandwidth) versus total photons detected without a filter. (**D, E**) Shielding tests (**D**) and quantification analysis of imaging signals (**E**).

To establish OI as a technique suitable for detecting a broad range of radionuclides, we surveyed the detectability of varying amounts of commonly used radionuclides by an IVIS Spectrum OI system. These radionuclides included β^+^ emitters (^18^F and ^64^Cu), β^−^ emitters (^131^I, ^90^Y, and ^177^Lu), and γ emitters [^111^In (also electron capture) and ^99m^Tc]. With the exception of ^99m^Tc, all tested radionuclides provided optical signals with good sensitivity with 1–5 min acquisition time (**Fig. 1A**). This encouraging result clearly verified our hypothesis. Under the conditions used in the studies, good signal-to-noise (S/N) ratios (>20 for ^18^F and >7 for ^131^I) were observed with 0.1 µCi of radioactivity for ^18^F and ^131^I, while 0.01 µCi of ^90^Y could be detected with a S/N of over 6. The plot of average radiance *vs.* radioactive intensity for these radionuclides (**Fig. 1B**) provided radionuclide's imaging sensitivity as *K* values of the slopes. Obviously, the pure β^−^ emitter ^90^Y has the highest sensitivity among all the radionuclides evaluated, and the detection sensitivity of ^18^F and ^131^I was ranked as 2^nd^ and 3^rd^, respectively. Finally, the pure γ emitter ^99m^Tc was undetectable even when high radioactivity (20 µCi) was applied, suggesting that OI signal was not caused by γ rays (**Fig. 1A**).

IVIS Spectrum system provides us with the ability to measure the OI imaging signal intensities at different wavelengths (from 490 to 850 nm). It was found that the total OI signals produced by these radionuclides were actually contributed by light with continuous wavelengths as monitored by the instrument. For the radionuclides tested, the percentages of optical signal intensity at different wavelengths vs. wavelength were shown in **Fig. 1C**. All of these radionuclides shared a similar distribution pattern, indicating the same mechanism for light production. Peak light intensity was observed at 490–540 nm, with slow reduction from 540 to 700 nm, remaining flat from 700 nm onwards (**Fig. 1C**). To further demonstrate that OI signals were not attributed to β^+^ or β^−^ particles or γ high energy radiation directly, white and black papers were used to cover the surface of the radioactive samples. It was found that the light signal could be significantly blocked by the coverage of a piece of white or black paper, and black paper also showed better OI signal shielding ability than white paper (**Fig. 1D** and **E**). Therefore, the optical signals detected were not from high energy β^+^ or β^−^ particle which can penetrate paper, but from the low energy optical photons that can be severely affected by the opacity of the covered material. Limited penetrability, a common disadvantage of OI, provided helpful information in determining the nature of signals detected by optical camera. It is worth noting that although optical signals can be significantly reduced by white paper, they are not completely blocked. The photons that penetrate through barrier can still be useful for *in vivo* imaging. The optical signal depends on many factors such as the amount of radioactivity, depth, tissue opacity, and tissue type.

### Phantom Imaging Study

**Figure 2:**
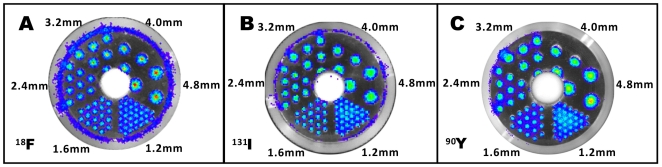
Phantom imaging studies with radioactive OI and PET. (**A–C**) Radioactive OI of ^18^F, ^131^I and ^90^Y phantoms.

To further explore the potential of using radioactive probes for OI, phantom imaging study was performed to determine the spatial resolutions of the three most sensitive radionuclides studied (^18^F, ^131^I and ^90^Y). For all three radionuclides, 1.2 mm spatial resolution was achieved (**Fig. 2A, B** and **C**). The detection resolution reported for PET and SPECT radionuclides is in the range of 1–2 mm [1]. Pure β^−^ emitter ^90^Y has very poor resolution (in cm range) when SPECT or γ cameras are used for imaging of its high energy radiation [7], [8], [9]. The results of phantom study demonstrated that the radioactive OI could be used for living subject imaging because of its high spatial resolution.

### *In Vivo* Radioactive OI with a β^+^ Emitter, ^18^F

**Figure 3:**
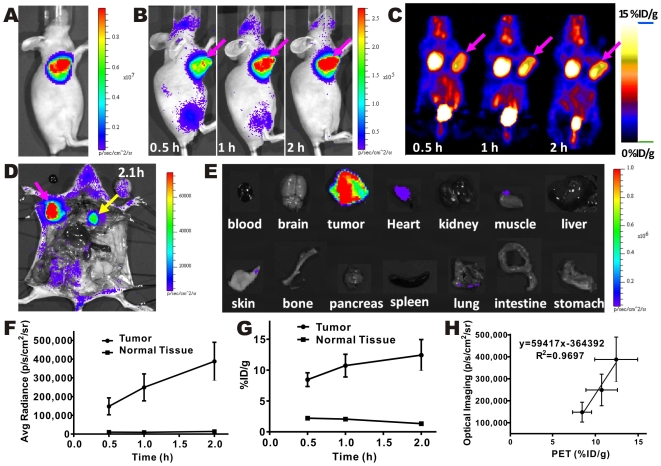
*In vivo* radioactive OI of [^18^F]FDG in comparison with microPET. (**A**) Bioluminescence image of a nude mouse bearing C6-FLuc tumor. (**B,C**) Radioactive OI and microPET imaging of a nude mouse bearing C6-FLuc tumor injected via tail vein with [^18^F]FDG at 0.5, 1, 2 h p.i. (**D,E**) Radioactive OI of the mouse after opening the thorax (**D**) and exposure the organs (**E**) at 2.1 h p.i. (**F–H**) Quantitative analysis of radioactive OI (**F**) and microPET (**G**) results and their correlation (**H**).

*In vivo* radioactive OI was first demonstrated by using two well-known PET probes, 2-deoxy-2-[^18^F]fluoro-D-glucose ([^18^F]FDG) and Na^18^F. [^18^F]FDG has been widely used for imaging of tumor metabolism [2], [10], [11], while Na^18^F accumulates in the bone [11]. In this study, a group of mice (n = 3) were implanted with firefly luciferase (FLuc) transfected rat C6 glioma cells (C6-FLuc). A day prior to the radioactive imaging, the mice were injected with luciferin and bioluminescent OI was performed to verify the presence of the tumor (**Fig. 3A**). [^18^F]FDG was injected in he mice bearing C6-FLuc glioma and imaged sequentially with IVIS Spectrum (**Fig. 3B**) and microPET (**Fig. 3C**). [^18^F]FDG was preferentially localized and was retained in C6-FLuc tumors, as clearly shown in both radioactive OI and microPET imaging technique (**Fig. 3B** and **C**). High signals from bladder and brain were also observed by both modalities. Heart, on the other hand, was hardly visible in the radioactive OI because of its location deep inside of the murine body. After we sacrificed and opened the thorax of the mice, the heart was easily identified (**Fig. 3D**, yellow arrow). To further characterize the distribution of [^18^F]FDG using radioactive OI, tumors and normal organs and tissues were removed from the sacrificed mice and subjected to optical imaging. High activity was mainly observed from the tumor and heart tissue samples (**Fig. 3E**).

Quantitative analysis of both radioactive OI and microPET images was performed. The OI intensities in the tumor and the normal tissue as a function of time for [^18^F]FDG was depicted in **Fig. 3F**, while accumulation of radioactivity in tumor and muscle over time was shown in **Fig. 3G**. It was found that radioactive OI and microPET imaging provided similar information with regards to [^18^F]FDG uptake and kinetics in tumor and muscle (**Fig. 3F** and **G**). The tumor uptake of [^18^F]FDG increased significantly (*P*<0.05) over time (from 0.5–2 h) for both modalities. Finally, in order to further evaluate the quantitative analysis data obtained from radioactive OI, correlation analysis was performed for the tumor uptakes obtained from the two imaging modalities. Good correlation was seen between the radioactive OI signal intensities and microPET images derived tumor uptakes (**Fig. 3H**, r^2^ over 0.90 for the analyses). This result illustrated the promise of radioactive OI as a tool for semi-quantitative analysis of the radioactive probe biodistribution.

**Figure 4:**
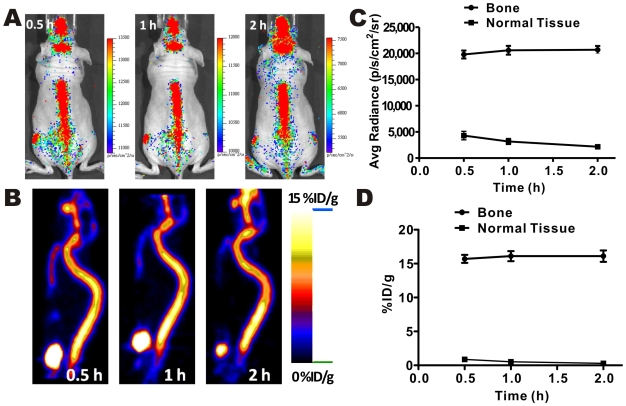
Dual modality Na^18^F imaging. Radioactive OI (**A**) and microPET imaging (**B**) of Na18F at 0.5, 1, 2 h after i.v. injection. Quantitative analysis of radioactive OI (**C**) and microPET (**D**) results.

The bone–seeking PET probe, Na^18^F, was also used for radioactive OI and microPET imaging studies to further confirm the use of radioactive probes for molecular OI. High and consistent imaging signals were observed in the bone structures (vertebral column, cranium, etc.) in both imaging modalities (**Fig. 4A, B**). The quantification analysis of the optical and PET images was also performed and the results were shown in **Fig. 4C** and **D**. Similar *in vivo* kinetics of Na^18^F were observed using both PET and radioactive OI.

### Radioactive OI with a β^−^ Emitter, ^131^I

**Figure 5:**
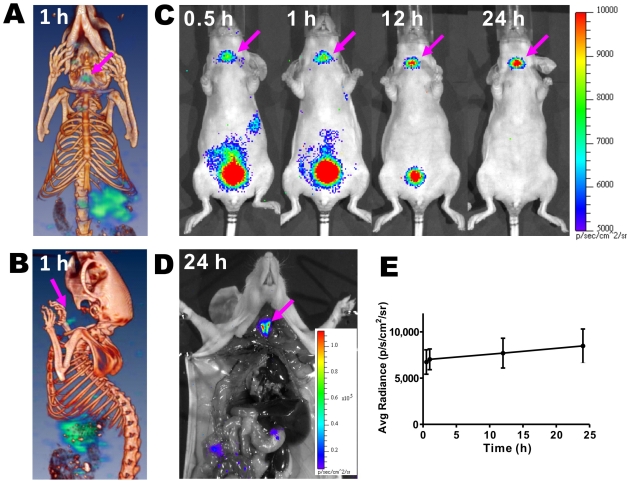
*In vivo* radioactive OI of Na^131^I compared to SPECT/CT imaging. Coronal (**A**) and sagittal (**B**) images of SPECT/CT imaging at 1 h after injection of Na^131^I probe. (**C**) Radioactive OI of a normal mouse at 0.5, 1, 12, 24 h after injection of Na^131^I via tail vein. (**D**) Radioactive OI of a normal mouse after opening the thorax 24 h post injection of Na^131^I. (**E**) Quantitative analysis (n = 3) of thyroid uptake of Na^131^I from radioactive OI results.

Iodine ion accumulates in the thyroid, and Na^123/131^I has been widely used for imaging thyroid function and sodium iodide symporter as well as for treating thyroid cancer [12], [13], [14]. We tested the SPECT probe, Na^131^I, for thyroid radioactive OI. Na^131^I SPECT/CT fusion images shown in **Fig. 5A** (coronal image) and **B** (sagittal image) clearly displayed the mouse bone anatomic structure (brown-red color) and the localization of the probe in the thyroid and abdomen region (green color, thyroid was indicated by an arrow). Radioactive OI showed the accumulation of the tracer in the thyroid and bladder as well, with signal observable at an earlier time point post injection (p.i.). The thyroid activity slowly increased, while the activity in the bladder cleared out at later time points (**Fig. 5C**). At the end of the OI study, the mice were sacrificed and the neck and chest areas were opened to expose the internal organs. The thyroid was clearly differentiated (**Fig. 5D**). Quantitative analysis of OI demonstrated accumulation and retention of Na^131^I in the thyroid (**Fig. 5E**).

### Radioactive OI with a Pure β^−^ Emitter, ^90^Y

**Figure 6:**
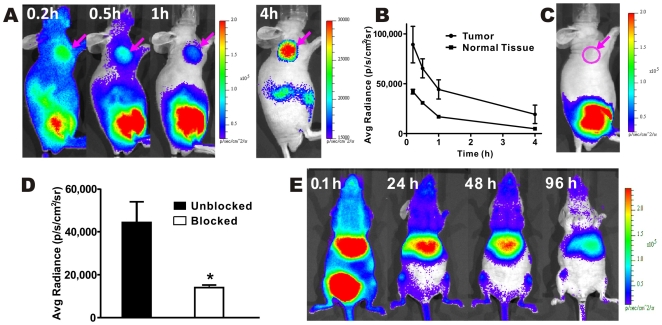
*In vivo* radioactive OI of ^90^Y-RGD-BBN and ^90^YCl_3_. Radioactive OI (**A**) and quantitative analysis (n = 3) (**B**) of ^90^Y-RGD-BBN in mice bearing PC3 tumor. (**C,D**) Receptor blocking studies of ^90^Y-RGD-BBN probe (at 1 h post-injection) using radioactive OI (**C**) and their quantification analysis (n = 3) (**D**). (**E**) Radioactive OI of ^90^YCl_3_ at various time points p.i.

Arg-Gly-Asp (RGD) coupled with bombesin (BBN) peptide (RGD-BBN) analogs were recently reported as heterodimeric peptides for dual targeting of integrin α_v_β_3_ and gastrin-releasing peptide receptor (GRPR). Radiolabeled (^64^Cu,^ 18^F, ^68^Ga, etc.) RGD-BBN peptides have been reported for PET imaging of human prostate cancer in a PC3 tumor mice model [15], [16], [17]. We labeled 1,4,7,10-tetraazacyclododecane-1,4,7,10-tetraacetic acid (DOTA) conjugated RGD-BBN with ^90^Y and tested ^90^Y-DOTA-RGD-BBN with radioactive OI. As shown in **Fig. 6A**, PC3 tumors were clearly visible with good tumor-to-background contrast at 1 and 4 h p.i. Also observed were a high accumulation of the probe in the kidneys at 0.2 h p.i., and in bladder at 0.5 and 1 h p.i. (**Fig. 6A**). For mice co-injected with a large excess of RGD and BBN peptides, the tumors were hardly visible on optical images at 1 h p.i. (**Fig. 6C**). Quantification analysis of optical images showed the kinetic of tumor targeting (**Fig. 6B**) and significantly lower tumor uptake for the probe co-injected with the cold analog at 1 h p.i. (*P*<0.05) (**Fig. 6D**).

Mice injected with ^90^YCl_3_ (1.8–2.0MBq each) were also optically imaged at different time points p.i. (**Fig. 6E**). It could be clearly seen that ^90^Y accumulated in the liver as well as in the bone. High activity in the bladder suggested the clearance route of this agent. The results obtained by the radioactive OI agree well with the previous reports which measured the in vivo behavior of ^90^YCl_3_ by biodistribution study [18].

## Discussion

Developing novel imaging techniques and instruments has been a major effort in the molecular imaging field. OI is a novel technique with many good characteristics such as high sensitivity, low-cost, ease of use, relatively high-throughput, and short acquisition time [1]. Recent advances in optical imaging instruments and molecular probes have made it an excellent tool for both small animal research and clinics. In this study, we demonstrate systematically that OI techniques can also be used to image visible and near-infrared light produced by radioactive materials. This would make OI a modality of choice for evaluation of bioluminescent, fluorescent and radioactive probes.

The success of using radionuclides for OI as demonstrated here is expected to have a major impact to the molecular imaging field. First, radioactive agents are traditionally studied by PET, SPECT or γ cameras, which are expensive, hard to maintain and not widely available to many researchers. Our results clearly show that the commercially available OI instrument can be used for studying radioactive probes possessing both β^+^ and β^−^-emitting radionuclides, as well as for bioluminescence and fluorescence probes. Considering much lower cost and wider accessibility of the OI instruments than that of PET and SPECT, radioactive probe development will likely be dramatically accelerated by using this approach. Optical imaging systems such as IVIS can image up to five animals simultaneously, while small animal PET and SPECT may only image one mouse at a time. The high throughput manner of OI equipments will also help to improve the speed of radioactive probe development.

Although OI is an important tool in animal research, its clinical applications have been severely hampered by very limited OI probes approved by the Food and Drug Administration (FDA). So far only iodocyanine green dye (IC-Green) has been approved for use in humans [19]. On the other hand, radioactive imaging has a longer history in biomedical imaging and has been widely used in clinics for the past several decades. Many FDA approved SPECT and PET probes including [^18^F]FDG have been developed for imaging different diseases and molecular targets. New applications for these radioactive probes may be developed in conjunction with radioactive OI techniques. The research presented here opens a new avenue for small animal imaging research, as well as for imaging patients in clinics.

In this research, we have evaluated three radionuclides (^18^F, ^131^I, and ^90^Y) for small animal radioactive OI because of their important roles in nuclear medicine. ^18^F is the most often used PET radionuclide [20], and ^131^I and ^90^Y are two most widely used radionuclides for radiotherapy [21], [22]. *In vivo* optical images with reasonable sensitivity can be quickly obtained for all three radionuclides. These encouraging results suggest that the radioactive OI can be a powerful tool for fast preliminary evaluation of ^18^F, ^131^I, and ^90^Y labeled compounds. This will be important for ^90^Y based agents development, since it has been relatively difficult to obtain *in vivo* information for a ^90^Y agent through non-invasive imaging method.

Compared to conventional fluorescence and bioluminescence imaging, radioactive OI has some unique properties. It has wide emission spectrum as demonstrated here, so that a radioactive probe can be monitored at different wavelengths. More importantly, radioactive OI does not require excitation light, which is a significant advantage over traditional OI. The radioactive OI signal generated by a radioactive probe is constitutive, which is very different from fluorescence and bioluminescence probes. The radioactive OI can be performed by monitoring spectral windows that differ from typical FLuc spectrum. Therefore, it is possible to perform BLI and radioactive OI in the same animal with proper emission filters. Radionuclides also generally emit low levels of light and are not expected to interfere with BLI.

It should be noted that although only β^+^ and β^−^ emitters were evaluated in this study, many radionuclides (α, β^+^, β^−^, electron capture, etc.) which emit charged particles are likely to be suitable for OI. The detection and imaging sensitivity depends on the physical properties of radionuclides, particularly their energy. As shown in this study, the radionuclide with higher energy generates stronger optical signals. Radioactive OI shares some common disadvantages as other OI techniques: limited tissue penetration and relatively poor quantification ability compared to PET and SPECT. However, subcutaneous tumor models and superficial disease models have been widely used in the medical research. For these models, our results indicate, radioactive OI is a suitable technique. Despite the stated disadvantages, OI techniques have advanced rapidly over the past couple of years. OI has evolved from a basic research tool to a modality which has great potential utility for patient imaging. For instance, fluorescence molecular tomography (FMT) and optical fiber based technology could potentially lead to deeper tissue imaging. These techniques may be very helpful for achievement of deeper tissue imaging with radioactive OI as well. Future research in radioactive OI would further accelerate the translation of OI into clinical application.

The optical signals detected could be originating from Bremsstrahlung or Cerenkov radiation. Bremsstrahlung radiation is a well-known physical phenomenon, which refers to the electromagnetic radiation produced by slowing down or deflection of charged particles (especially electrons) in the Coulomb fields of atomic nuclei [23], [24] In Cerenkov radiation, optical signal is emitted when a charged particle travels through an insulator at a speed greater than the speed of light in that medium [25], [26]. Both of them have continuous energy spectra in optical and NIR range and are generated by radioactive disintegration of a charged particle [23], [24], [25], [26]. The recent publication on radioactive OI by Robertson et al. strengthens our data. They propose Cerenkov radiation as the origin of the optical signals generated from positron emitting nuclides [6]. Cerenkov radiation was supported by the following data: (1) The obtained radioactive optical spectrum and (2) the increased light output as the refractive index of medium increases. Initially, we assigned generation luminescence signal to Cerenkov radiation as well. We obtained similar results which optical signal strength was enhanced with increasing refractive indexes of medium (**Fig. S1**). But the spectra obtained from IVIS system (**Fig. 1**) were quite different from the calculated Cerenkov emission in literature [26]. Radioluminescence intensities, measured with a Fluoro Max-3 spectrofluorometer (Jobin Yvon Inc., Edison, NJ), were consistent with the spectra obtained from the IVIS system (**Fig. S2**). Moreover, water has a refractive index of 1.332. The lower energy threshold for the emission of Cerenkov radiation in water is thus calculated to be 263 KeV [26]. However, radioactive optical signals could also be observed for ^111^In, which emits particles with the energy below the theoretical threshold. All these data imply that other mechanisms such as Bremsstrahlung radiation may also contribute to the radioactive OI. It is unclear as for the role of Bremsstrahlung radiation compared to Cerenkov. The mechanism of radioactive OI still needs to be further investigated.

## Materials and Methods

### Materials

Na^131^I, Na^125^I, ^90^YCl_3_, and ^111^InCl_3_ were purchased from Perkin Elmer (Waltham, MA). Sodium pertechnetate (Na^99m^TcO_4_) was obtained from GE Healthcare Nuclear Pharmacies (Sunnyvale, CA), ^177^LuCl_3_ and ^64^CuCl_2_ was provided by the University of Missouri Research Reactor (Columbia, MO) and the Department of Medical Physics, University of Wisconsin at Madison (Madison, WI), respectively. [^18^F]FDG and Na^18^F were produced by Radiochemistry Facility at Stanford University (Stanford, CA). Heterodimer peptide DOTA-RGD-BBN was provided by Dr. Xiaoyuan Chen as reported recently [15], [16], [17]. All other standard synthesis reagents were purchased from Sigma-Aldrich Chemical Co. (St. Louis, MO). Human prostate cancer cell line PC3 was obtained from American Type Culture Collection (Manassas, VA). Rat glioma cell C6-FLuc was from Dr. Gambhir's laboratory. Female athymic nude mice (*nu/nu*), obtained from Charles River Laboratories, Inc. (Cambridge, MA) were at 4–6 weeks of age. All instruments including electrospray ionization mass spectrometry (ESI-MS), reverse phase high performance liquid chromatography (RP-HPLC), PET dose calibrator, and tumor cell lines are the same as described in our previous publication [27].

### Tumor Models

All animal studies were carried out in compliance with Federal and local institutional rules for the conduct of animal experimentation. C6-FLuc cells were cultured in DMEM medium supplemented with 10% fetal bovine serum (FBS) and 1% penicillin-streptomycin (Invitrogen Life Technologies, Carlsbad, CA). PC3 cells were cultured in F-12K medium with 2 mM L-glutamine supplemented with 10% FBS and 1% penicillin-streptomycin. All the cell lines were maintained in a humidified atmosphere of 5% CO_2_ at 37°C, with the medium changed every other day. A 75% confluent monolayer was detached with trypsin and dissociated into a single cell suspension for further cell culture. Approximately 1×10^6^ C6-FLuc or PC3 cells suspended in PBS were implanted subcutaneously in the flanks of nude mice. Tumors were allowed to grown to a size of 500 to 750 mg (2–3 weeks), and the tumor bearing mice were subjected to in vivo imaging and biodistribution studies.

### *In Vivo* Bioluminescence Imaging

The mice bearing subcutaneous C6-FLuc tumors were anesthetized in a chamber filled with 2% isofluorane in oxygen, and then transferred to the light-tight chamber of the IVIS Spectrum small animal imaging system (Caliper Life Sciences, Hopkinton, MA). D-Luciferin was injected intraperitoneally and the images were acquired at 10 min after injection.

### Radioactive Optical Imaging

Radioactive OI was performed with an IVIS Spectrum system (Caliper Life Science, Hopkinton, MA). For all *in vivo* and *in vitro* studies, radionuclides were diluted in phosphate-buffered saline (PBS; Invitrogen). Aliquots of 300 µL PBS solution with the radionuclide were placed in each well of flat bottom 96-wells black plates (Nunc, Naperville, IL) for *in vitro* studies. Wavelength-resolved spectral imaging was carried out using an 18-set narrow bands emission filters set (490–850 nm). Animals were placed in a light-tight chamber under isofluorane anesthesia. Each acquisition, with or without filters, took 1–5 min for all studies. Images were acquired and analyzed using Living Image 3.0 software (Caliper life sciences, Hopkinton, MA). Quantification of radioactive OI images was corrected in accordance with the radionuclides' respective physical decay properties. The dorsal skin area was used as normal tissue representing muscle tissue for *in vivo* studies. Optical signal was normalized to photons per second per centimeter square per steradian (p/s/cm^2^/sr). Identical setting was used for *in vitro* Radioactive OI. For *in vivo* imaging study, normal mice or mice bearing either C6-FLuc or PC3 (n = 3 for each imaging probe) were injected via tail vein with Na^18^F (5.3–5.7 MBq, 140–150 µCi), [^18^F]FDG (10.4–11.3 MBq, 280–305 µCi), Na^131^I (2.2–2.3 MBq, 59–61 µCi), ^90^YCl_3_ (1.8–2.0 MBq, 48–54 µCi) or ^90^Y-RGD-BBN (specific activity: 63 µCi/µg, 2.6–3.3 MBq, 70–90 µCi). For [^18^F]FDG imaging study, the mice were fasted over night prior to the experiment.

### Phantom Studies

A Micro Deluxe phantom with different sized hot-rod inserts (diameters, 1.2, 1.6, 2.4, 3.2, 4.0, and 4.8 mm) arranged in 6 segments (Data Spectrum Corp. Hillshorough, NC) was used for phantom studies. The phantom was filled with 10 mL PBS with [^18^F]FDG (2.6 MBq, 70 µCi), Na^131^I (2.2 MBq, 60 µCi) or ^90^YCl_3_ (1.9 MBq, 50 µCi). Radioactive OI was performed with an exposure time of 1–3 min without filters. Images were processed using Living Image 3.0 software.

### PET Imaging

Small-animal microPET imaging of tumor-bearing mice was performed on a small-animal PET R4 rodent model scanner (Siemens Medical Solutions USA, Inc., Knoxville, TN). A group of mice (n = 3) bearing C6-FLuc were injected with [^18^F]FDG (10.4–11.3 MBq, 280–305 µCi) *via* tail vein. At 0.5, 1 and 2 h p.i., the mice were anesthetized with 2% isofluorane (Aerrane' Baxter, Deerfield, IL), and placed in the prone position and near the center of the field of view (FOV) of small-animal PET scanner. Three-min static scans were obtained and the images were reconstructed by a two-dimensional ordered subsets expectation maximum (OSEM) algorithm. No background correction was performed. The method for quantification analysis of the images was the same as reported previously [27].

### SPECT/CT Imaging

Images were obtained on an Imtek microCAT II/SPECT system (Imtek, Inc. Knoxville, TN) at 1 h after injection of 2.2–2.3 MBq ^131^I through tail vein. Computed tomography images were acquired immediately after the SPECT scan. Co-registration of CT and SPECT images was done using Amira software (Amira 3.1, Mercury Computer Systems GmbH, Berlin, Germany). No X-ray contrast agent was used in this study.

## Supporting Information

Figure S1 Radioactive optical signals increase in medium with higher refractive indexes. Light outputs of 7 µCi ^131^I in various media with different refractive indexes were imaged by IVIS spectrum system in 96-well plates. (Refractive indexes: water, 1.333; 24% Glycerol, 1.363; 46% Glycerol, 1.392; 67% Glycerol, 1.423; 86% Glycerol, 1.452; DMSO, 1.479; DMF, 1.431.)
(0.22 MB TIF)

Figure S2 Radioactive OI spectra of ^90^Y, ^18^F and ^131^I by Fluoro Max-3. The spectra are consistent with those obtained by IVIS spectrum imaging system.
(0.37 MB TIF)
