# Supplementary figures and images for: Molecular Optical Imaging with Radioactive Probes
Source: PLoS One. 2010 Mar 1;5(3):e9470. doi: 10.1371/journal.pone.0009470 (PMC2830426; doi:10.1371/journal.pone.0009470)

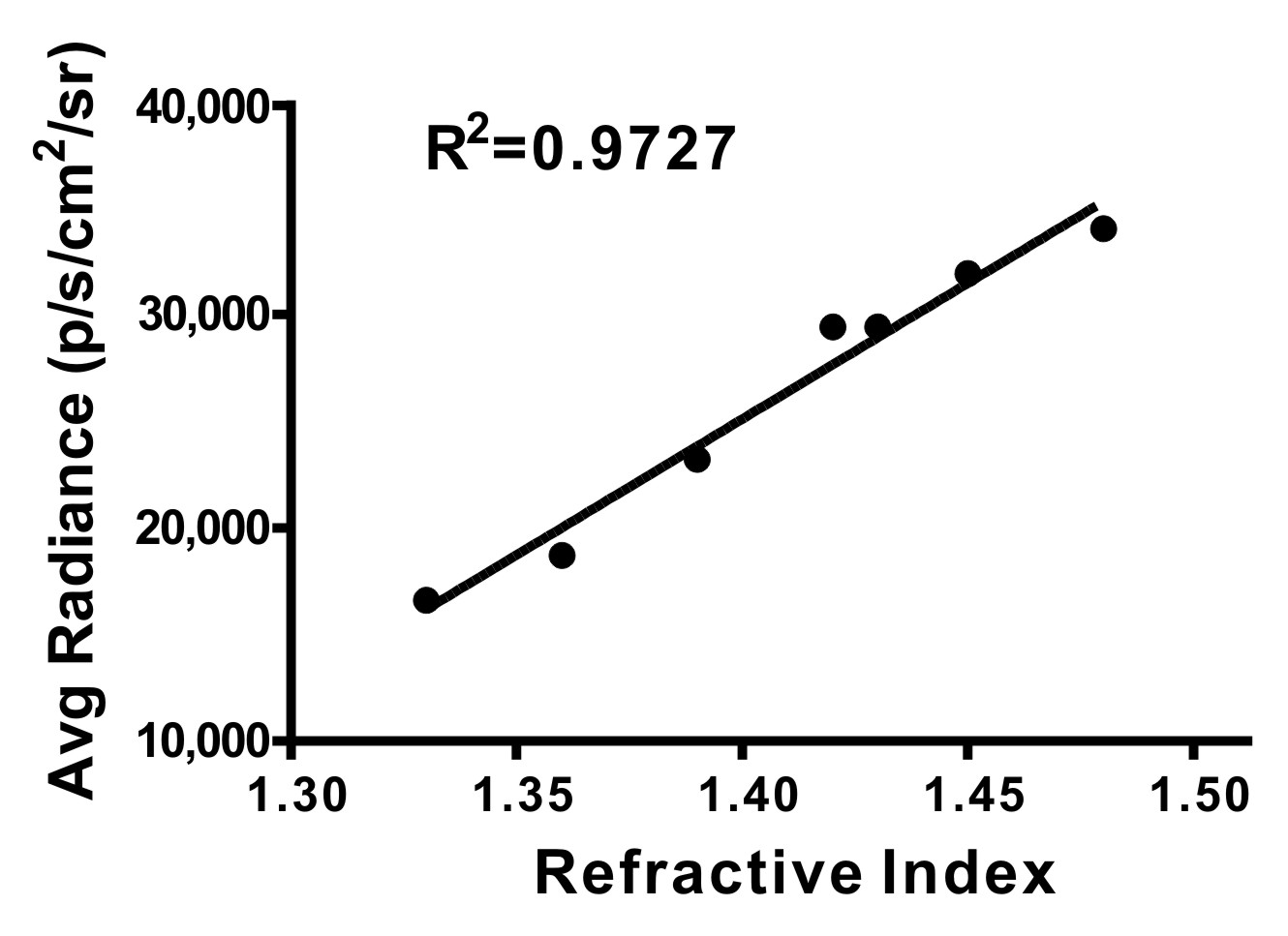

Supplement: Figure S1 — Radioactive optical signals increase in medium with higher refractive indexes. Light outputs of 7 µCi 131I in various media with different refractive indexes were imaged by IVIS spectrum system in 96-well plates. (Refractive indexes: water, 1.333; 24% Glycerol, 1.363; 46% Glycerol, 1.392; 67% Glycerol, 1.423; 86% Glycerol, 1.452; DMSO, 1.479; DMF, 1.431.) (0.22 MB TIF) [file pone.0009470.s001.tif]

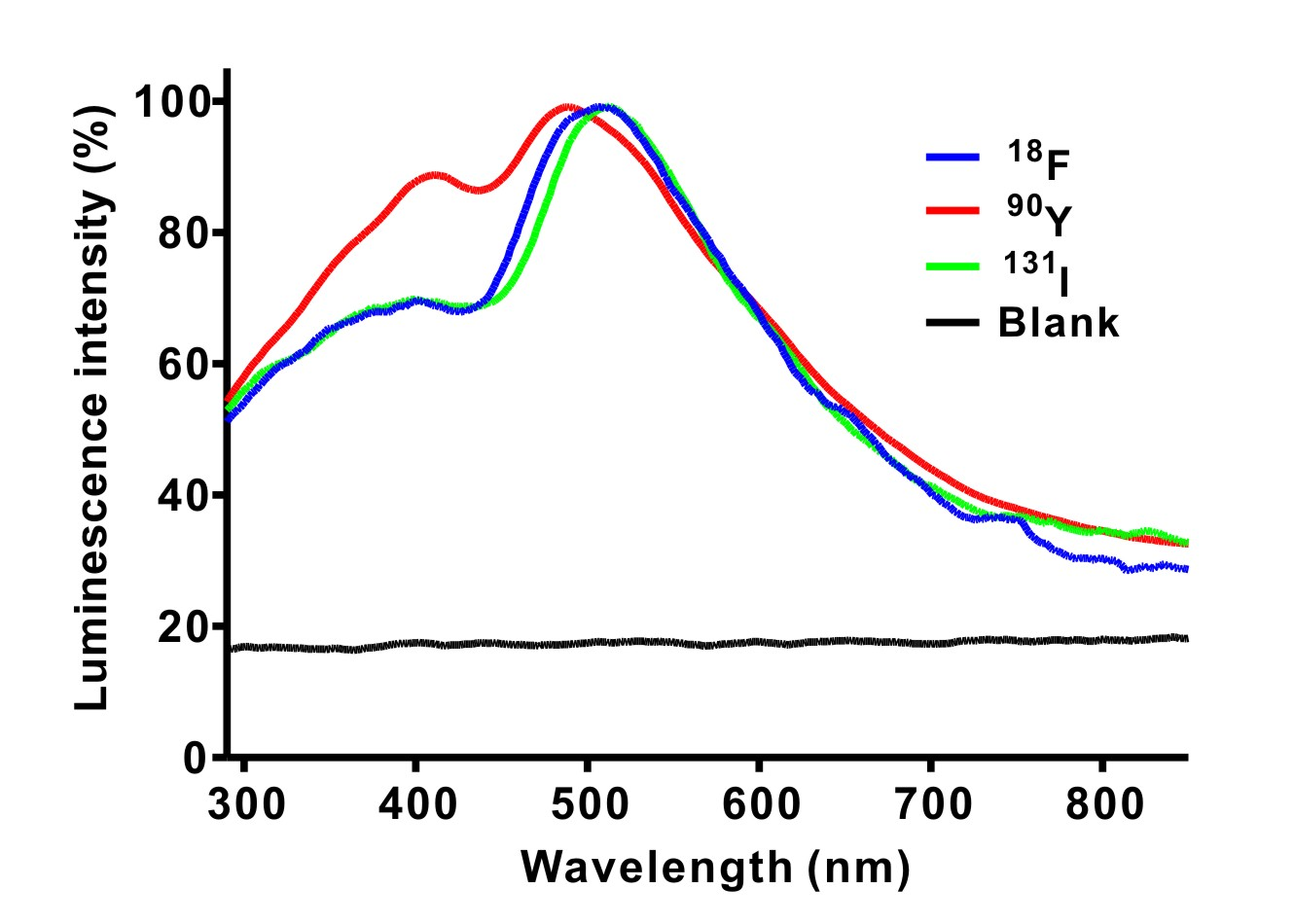

Supplement: Figure S2 — Radioactive OI spectra of 90Y, 18F and 131I by Fluoro Max-3. The spectra are consistent with those obtained by IVIS spectrum imaging system. (0.37 MB TIF) [file pone.0009470.s002.tif]
